# Supplementary material for: The role of human factors in paramedics’ clinical judgement – A modified Delphi study
Source: PLoS One. 2025 Sep 15;20(9):e0332311. doi: 10.1371/journal.pone.0332311 (PMC12435673; doi:10.1371/journal.pone.0332311)
Supplement: S1 File — (PDF) [file pone.0332311.s001.pdf]

S1 file. Literature review process to identify relevant human factors in clinical judgement in EMS settings.

Search strategy and terms used:
ALL(("emergency medical service*") OR ("paramedic*") OR ("prehospital") OR ("pre-hospital") OR ("out of hospital") OR ("ambulance") OR ("ambulance crew") OR ("EMT") OR ("emergency medical technician") OR ("emergency care practitioner") OR ("emergency practitioner") OR ("prehospital nurse*") OR ("pre-hospital nurse*")) AND ALL(("human factor*") OR ("ergonomics")) AND ALL(("decision making") OR ("decision-making") OR ("clinical decision making") OR ("clinical judgment") OR ("clinical judgement") OR ("clinical reasoning"))

- peer-reviewed
- published in 2012-2023,
- language English
- academic journals
- full text available or access to full text via the Library of University of Helsinki

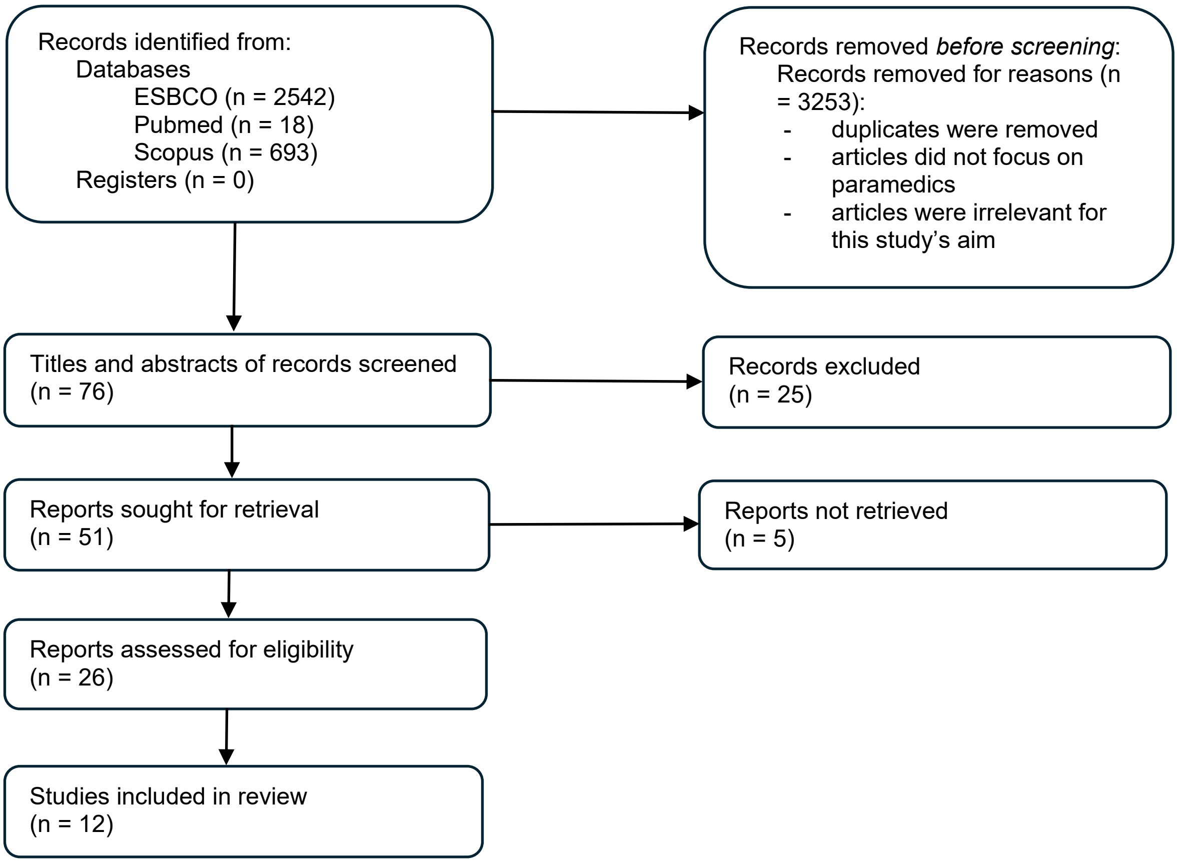

Included studies:

- Bijani M, Abedi S, Karimi S, Tehranineshat B. Major challenges and barriers in clinical decision-making as perceived by emergency medical services personnel: a qualitative content analysis.
- Brandling J, Kirby K, Black S, Voss S, Benger J. Emergency medical service provider decision-making in out of hospital cardiac arrest: an exploratory study.
- Groombridge CJ, Kim Y, Maini A, Smit DV, Fitzgerald MC. Stress and decision-making in resuscitation: A systematic review.

- Hetherington J, Jones I. What factors influence clinical decision making for paramedics when attending to paediatric emergencies in the community within one ambulance service trust?
- Leemeyer A-MR, Van Lieshout EMM, Bouwens M, Breeman W, Verhofstad MHJ, Van Vledder MG. Decision making in prehospital traumatic cardiac arrest; A qualitative study.
- O'Hara R, Johnson M, Siriwardena AN, Weyman A, Turner J, Shaw D, et al. A qualitative study of systemic influences on paramedic decision making: care transitions and patient safety.
- Ozkaynak M, Dolen C, Dollin Y, Rappaport K, Adelgais K. Simulating Teamwork for Better Decision Making in Pediatric Emergency Medical Services.
- Penney G, Launder D, Cuthbertson J, Thompson MB. Threat assessment, sense making, and critical decision-making in police, military, ambulance, and fire services.
- Perona M, Rahman MA, O’Meara P. Paramedic judgement, decision-making and cognitive processing: A review of the literature.
- Poranen A, Kouvonen A, Nordquist H. Perceived human factors from the perspective of paramedics – a qualitative interview study.
- Reay G, Rankin JA, Smith-Macdonald L, Lazarenko GC. Creative adapting in a fluid environment: An explanatory model of paramedic decision making in the pre-hospital setting.
- Safi-Keykaleh M, Khorasani-Zavareh D, Ghomian Z, Bohm K. A model to explain the challenges of emergency medical technicians' decision making process in emergency situations: a grounded theory.
