# Supplementary material for: The role of human factors in paramedics’ clinical judgement – A modified Delphi study
Source: PLoS One. 2025 Sep 15;20(9):e0332311. doi: 10.1371/journal.pone.0332311 (PMC12435673; doi:10.1371/journal.pone.0332311)
Supplement: Attachment — Submitted filename: Response to reviewers.docx [file pone.0332311.s003.docx]

**Dear Dr. David Wampler,**

We were pleased that you gave us an opportunity to submit a revised draft of our manuscript: "The role of human factors in paramedics’ clinical judgement – A modified Delphi study”. We are grateful for the insightful comments provided by you and the reviewer. Those comments dealt with important issues, leading us to revise the manuscript in accordance with those comments.

Please see below for a point-by-point response to the concerns raised by you and the reviewer in blue. We have highlighted revisions in the text in yellow. Line numbers below refer to the marked copy; we have additionally uploaded a clean copy.

We hope that our response adequately addresses all the points raised and we look forward to hearing from you.

Sincerely,

Anna Poranen, MSc, on behalf of all the authors

Role of funder statement

Open access was funded by Helsinki University Library. The funder had no role in study design, data collection and analysis, decision to publish, or preparation of the manuscript.

Comments and concerns:

1. Please ensure that your manuscript meets PLOS ONE's style requirements, including those for file naming. The PLOS ONE style templates can be found at

<https://journals.plos.org/plosone/s/file?id=wjVg/PLOSOne_formatting_sample_main_body.pdf>

<https://journals.plos.org/plosone/s/file?id=ba62/PLOSOne_formatting_sample_title_authors_affiliations.pdf>

**Our response 1: We have corrected file names and checked that our manuscript meets PLOS ONE’s style requirements.**

2. Thank you for stating the following financial disclosure:

Open access funded by Helsinki University Library

Please state what role the funders took in the study. If the funders had no role, please state: "The funders had no role in study design, data collection and analysis, decision to publish, or preparation of the manuscript."

If this statement is not correct you must amend it as needed.

Please include this amended Role of Funder statement in your cover letter; we will change the online submission form on your behalf**.**

**Our response 2: We have now added the funder’s role as follows: “Open access was funded by Helsinki University Library. The funder had no role in study design, data collection and analysis, decision to publish, or preparation of the manuscript.”**

3. We note that you have indicated that there are restrictions to data sharing for this study. For studies involving human research participant data or other sensitive data, we encourage authors to share de-identified or anonymized data. However, when data cannot be publicly shared for ethical reasons, we allow authors to make their data sets available upon request. For information on unacceptable data access restrictions, please see http://journals.plos.org/plosone/s/data-availability#loc-unacceptable-data-access-restrictions.

Before we proceed with your manuscript, please address the following prompts:

a) If there are ethical or legal restrictions on sharing a de-identified data set, please explain them in detail (e.g., data contain potentially identifying or sensitive patient information, data are owned by a third-party organization, etc.) and who has imposed them (e.g., a Research Ethics Committee or Institutional Review Board, etc.). Please also provide contact information for a data access committee, ethics committee, or other institutional body to which data requests may be sent.

b) If there are no restrictions, please upload the minimal anonymized data set necessary to replicate your study findings to a stable, public repository and provide us with the relevant URLs, DOIs, or accession numbers. Please see http://www.bmj.com/content/340/bmj.c181.long for guidelines on how to de-identify and prepare clinical data for publication. For a list of recommended repositories, please see https://journals.plos.org/plosone/s/recommended-repositories. You also have the option of uploading the data as Supporting Information files, but we would recommend depositing data directly to a data repository if possible.

Please update your Data Availability statement in the submission form accordingly.

**Our response 3: We have now updated our Data Availability statement as follows:**

**The datasets generated and/or analysed in the current study are not publicly available because of privacy concerns. The informed consent indicated that the collected data would be used solely for this study, and no prior consent was obtained from the Delphi panelists to share their responses beyond the research team. While the content of the questionnaire and the response options are described in this paper, full transcripts of the open-ended responses have not been provided in order to preserve participant confidentiality. However, data access requests may be made to the corresponding author (anna.e.poranen@helsinki.fi) upon reasonable request, provided that participant confidentiality can be maintained. Alternatively, requests may also be directed to the University of Helsinki Research Ethics Committee in the Humanities and Social and Behavioural Sciences (eettinen-toimikunta@helsinki.fi).**

4. Please remove all personal information, ensure that the data shared are in accordance with participant consent, and re-upload a fully anonymized data set.

Note: spreadsheet columns with personal information must be removed and not hidden as all hidden columns will appear in the published file.

**Our response 4: Please see our previous response.**

5. If the reviewer comments include a recommendation to cite specific previously published works, please review and evaluate these publications to determine whether they are relevant and should be cited. There is no requirement to cite these works unless the editor has indicated otherwise.

**Our response 5: Thank you for this guideline, we have acted accordingly.**

6. Please review your reference list to ensure that it is complete and correct. If you have cited papers that have been retracted, please include the rationale for doing so in the manuscript text, or remove these references and replace them with relevant current references. Any changes to the reference list should be mentioned in the rebuttal letter that accompanies your revised manuscript. If you need to cite a retracted article, indicate the article’s retracted status in the References list and also include a citation and full reference for the retraction notice.

**Our response 6: We reviewed our reference list in accordance with the instructions. In addition, we have added two new references, please see Our response 13.**

Reviewers' comments:

Reviewer #1: The authors continue their work amidst a deeply needed task: seeking to understand the significant factors that drive or hinder our paramedic colleagues in the field. If we can understand the highest-leverage issues, we’re more likely to serve as a positive force in medical direction, training, or administrative tasks.

Paper flowed well; I enjoyed reading it, and suspect others will also.

**Our response 7: Thank you, we appreciate your feedback and time given to our article.**

Please forgive attempts to note minor editing/formatting issues that would be the purview of the editor(s); I assume they’re quite busy and offer my observations as I see them.

Major comment(s):

Results, page 9 of manuscript, lines 220-226, also discussion, page 23, lines 481-492: (Bottom line up front, please add to strengths/limitations) One of the only criticisms I had about this excellent work pertains to selection bias/healthy volunteer effect/survivorship bias – the fact that the ‘expert’ participants were largely self-selected, and skewed (possibly?) older (acknowledging the requirement to have at least 5 years in EMS, and the fact that one had to have a bachelor’s degree to begin with) means there won’t be as many younger people available to participate – but perhaps more importantly, in Wald statistic fashion, we only know what was most important to 47-ish people willing to speak up about it. Those who left the profession earlier in the career, or those who elected not to enroll, there’s no reason not to think those people wouldn’t prove systematically different to the enrollees. All those words said, ultimately the work reveals themes deemed important by those who remained committed to the career and motivated enough to respond, allowing subsequent lines of inquiry.

**Our response 8: Thank you for this important comment. Prior to data collection, we agreed that a minimum of five years work experience would ensure that experts have sufficient clinical competence of EMS to guarantee the credibility of this study. That is why we chose not to include paramedics who had only recently graduated. However, it is important to acknowledge that there may be potential bias of self-selection.**

**We have now clarified this in the Strengths and limitations section, and it now reads:**

**“Another potential bias may be self-selection as participation may have predominantly included paramedics who were particularly motivated to share their views on the topic. This may have influenced the representativeness of the findings.” (Lines 497-500)**

General comment: Methods changed in the middle of the work – no adequate consensus manifested, so the threshold was (appropriately) “modified;” it goes like that sometimes. Just ensure the methods section adequately reflects that adjustment; currently it’s something of a surprise in the results section (though alluded to, somewhat, with mention of medians in lines 190-193).

**Our response 9: Thank you for this important suggestion. We have now revised the text in the Methods -section. It now reads:**

**“While the initial plan was to analyse consensus based on percentage agreement, the analysis approach was adjusted after Round 2 and medians and interquartile ranges (IQR) were calculated allowing a more nuanced assessment of consensus in Likert-scale responses.” (Lines 191-193)**

Minor comment(s):

Introduction section, page 3 of manuscript, line 54: Consider Oxford comma following “under time pressure”

**Our response 10: We have now added an Oxford comma: “Paramedics work in challenging circumstances, under time pressure, and have to treat and deal with a wide range of medical and social problems and make quick and effective decisions.” (Line 54)**

Introduction “ “, line 57: Consider comma after “social care services,” alternatively a semicolon, or nothing as so desired

**Our response 11: We have now added comma after “social care services” and it now reads:**

**“In many rapidly ageing societies, including Finland, emergency medical services (EMS) have increasingly started to complement other health and social care services, and paramedics often assess and treat patients in their homes in a more comprehensive way [3, 4].” (Line 57)**

**References:**

**3. Ebben RHA, Vloet LCM, Speijers RF, Tönjes NW, Loef J, Pelgrim T, et al. A patient-safety and professional perspective on non-conveyance in ambulance care: a systematic review. Scand J Trauma Resusc Emerg Med. 2017;25(1):71.**

**4. Paulin J. Non-conveyance and patient safety in prehospital emergency care [Dissertation]: University of Turku; 2022.**

Methods, page 7 of manuscript, line 168: (Feb 2024) seemed out of place – was this when the data was analyzed, or the version number of SPSS used was released? Recommend either “IBM SPSS Statistics for Windows, Version XX.0 (IBM Corp., Armonk, NY)” or swap to the specific release date in parentheticals… assuming of course that I’ve correctly parsed your intent

**Our response 12: Thank you for pointing this out. We have now adjusted the text to better reflect the intended meaning. It now reads:**

**“Descriptive statistics were calculated using IBM SPSS Statistics for Windows, version 28.0 (IBM Corp., Armonk NY) and the analysis was conducted in February 2024.” (Lines 168-170)**

Discussion, page 21, lines 439-440: I would contend that the risks of violence against EMS personnel are well established (citations follow); while it’s not necessarily in the scope of the paper to drive the point of prevalent violence against our prehospital colleagues, I’d suggest we can move beyond stating the violence risks “may” be common, or merely a feeling – they're quite substantial, at least in places it’s been looked at.

Workplace violence prevention for paramedicine clinicians: Proposed U.S. bill addresses a data collection gap. (2023, April 18). Journal of Emergency Medical Services. https://www.jems.com/ems-management/legal-issues/workplace-violence-prevention-for-paramedicine-clinicians-proposed-u-s-bill-addresses-a-data-collection-gap/ (Salient reference therein: Maguire BJ, Al Amiry A, O’Neill BJ. Abstract 3278 – An examination of injuries and illnesses among paramedicine clinicians; using U.S. Department of Labor data to identify risks and compare risks to other occupational groups. American Public Health Association Conference; 2022; Boston, MA.)

Rahimi, M., Fallah Kharmandar, P., Aghababaeian, H., Sotoudeh, F., Afshari, A., & Hasani-Sharamin, P. (2020). Prevalence of workplace violence types against personnel of emergency medical services (EMS): A systematic review and meta-analysis. Health Promotion Perspectives, 10(1), 28–35. https://doi.org/10.15171/hpp.2020.06

McGuire, S. S., Gazley, B., Crowe, R. P., Bentley, M. A., O’Neil, C., & Koller, C. A. (2024). Workplace violence against emergency medical services (EMS): A prospective 12-month cohort study evaluating prevalence and risk factors within a large, multistate EMS agency. Prehospital Emergency Care. Advance online publication. <https://doi.org/10.1080/10903127.2024.2411020>

**Our response 13: Thank you for this comment, this is important to clarify. We have now revised the text as follows:**

**“This is in line with previous studies showing that risks to occupational safety and the risk of violence against paramedics are common [35, 36]. For example, systematic review by Murray et al. [35] shows that 57-93% of paramedics have experienced verbal and/or physical violence at least once during their career.” (Lines 443-446)**

**References:**

**35. Murray RM, Davis AL, Shepler LJ, Moore-Merrell L, Troup WJ, Allen JA, et al. A Systematic Review of Workplace Violence Against Emergency Medical Services Responders. New Solut. 2020;29(4):487-503.**

**36. McGuire SS, Bellolio F, Buck BJ, Liedl CP, Stuhr DD, Mullan AF, et al. Workplace Violence Against Emergency Medical Services (EMS): A Prospective 12-Month Cohort Study Evaluating Prevalence and Risk Factors Within a Large, Multistate EMS Agency. Prehospital Emergency Care. 2024(1-8).**
